# Supplementary material for: Evaluating the feasibility of 12-lead electrocardiogram reconstruction from limited leads using deep learning
Source: Commun Med (Lond). 2025 Apr 25;5:139. doi: 10.1038/s43856-025-00814-w (PMC12032410; doi:10.1038/s43856-025-00814-w)
Supplement: Supplementary file 2 — Supplementary Materials [file 43856_2025_814_MOESM2_ESM.pdf]

Supplementary Material

Supplementary Table S1: Root mean squared error (RMSE) per lead comparing real and electrocardiograms reconstructed with UNet

	Lead I	Lead II	V1	V2	V3	V4	V5	V6
1-lead reconstruction (μV)	-	79	76	136	136	120	96	79
2-lead reconstruction (μV)	-	-	72	134	135	110	82	64
SD* real	122	136	145	239	231	235	212	166

Average root mean squared error (RMSE) for every lead of the reconstructed electrocardiograms. The results were derived from the UNet models trained using both 1-lead and 2-lead configurations.

*SD = standard deviation

Supplementary Figure S1: Relationship between amplitudes in lead II and lead V3 for R-waves and T-waves for real and generated electrocardiogram (ECG) data

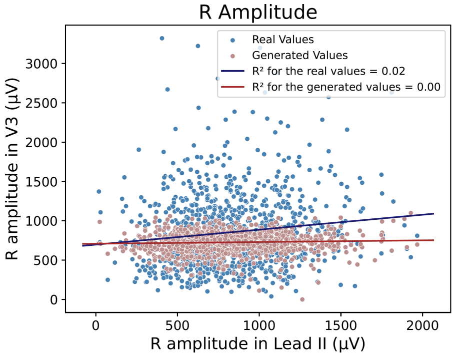

(a) 1-lead reconstruction

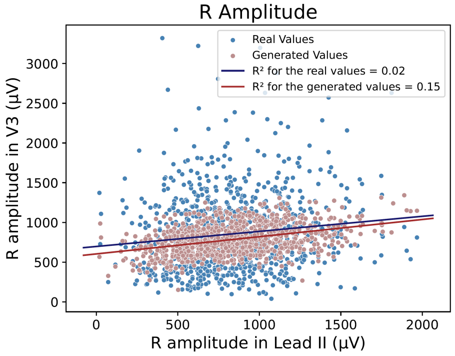

(b) 2-leads reconstruction

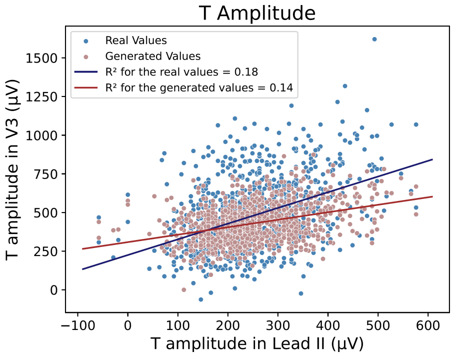

(c) 1-lead reconstruction

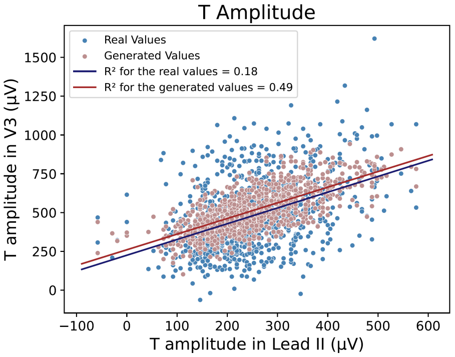

(d) 2-leads reconstruction

Relationship between Amplitudes in lead II and lead V3 for R-waves (a) and (b) and T-waves (c) and (d). Panels (a) and (c) show results from the 1-lead reconstruction, while panels (b) and (d) present results from the 2-lead reconstruction (n = 952 independent samples).

Each point represents an individual sample, with real values shown in blue and generated values in red. The lines indicate linear regression fits for the real (blue) and generated (red) data. The coefficient of determination (R^2) is reported for both cases. All amplitudes are measured in microvolts (µV).

Supplementary Table S2: Comparison of R^2 correlation coefficients for amplitudes in linear model vs. GAN-generated electrocardiogram data

ECG feature	Lead	GAN 1-lead	LM* 1-lead	GAN 2-lead	LM* 2-lead
R amplitude	V1	0.15	0.11	0.24	0.33
	V2	0.08	0.24	0.13	0.21
	V3	0.06	0.19	0.09	0.22
	V6	0.04	0.23	0.24	0.71
T amplitude	V1	0.10	0.28	0.06	0.28
	V2	0.18	0.32	0.18	0.32
	V3	0.24	0.36	0.30	0.46
	V6	0.44	0.61	0.65	0.83
S amplitude	V1	0.11	0.26	0.30	0.26
	V2	0.04	0.19	0.14	0.19
	V3	0.05	0.28	0.15	0.37
	V6	0.14	0.47	0.47	0.71
ST amplitude	V1	0.03	0.24	0.03	0.34
	V2	0.34	0.29	0.39	0.29
	V3	0.51	0.44	0.53	0.48
	V6	0.48	0.53	0.62	0.78

Contrasting R^2 correlation coefficients for R, S, T, and ST amplitudes, between linear model- and GAN-generated data across leads V1, V2, V3, and V6. The following equations were used for the linear models: y1 = a * (average RR interval) + b * (R amplitude in lead I); y2 = a * (average RR interval) + b * (R amplitude in lead I) + c * (R amplitude in lead II).

*LM = linear model

Supplementary Figure S2: Visualization of real and reconstructed electrocardiogram (ECG)

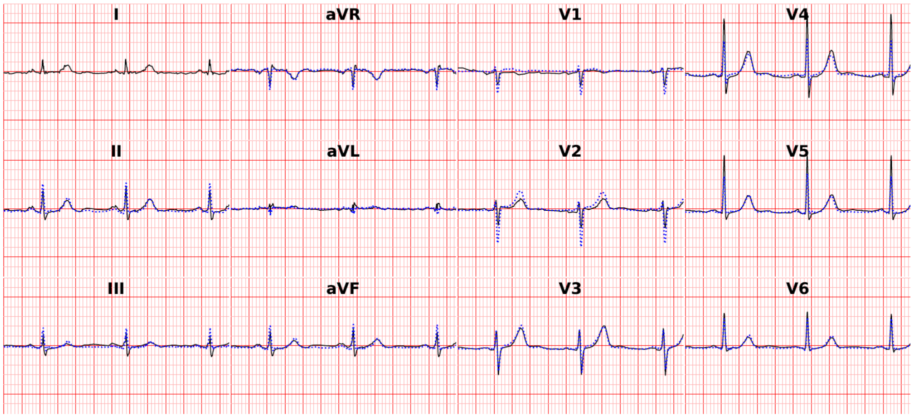

(a) 1-lead reconstruction

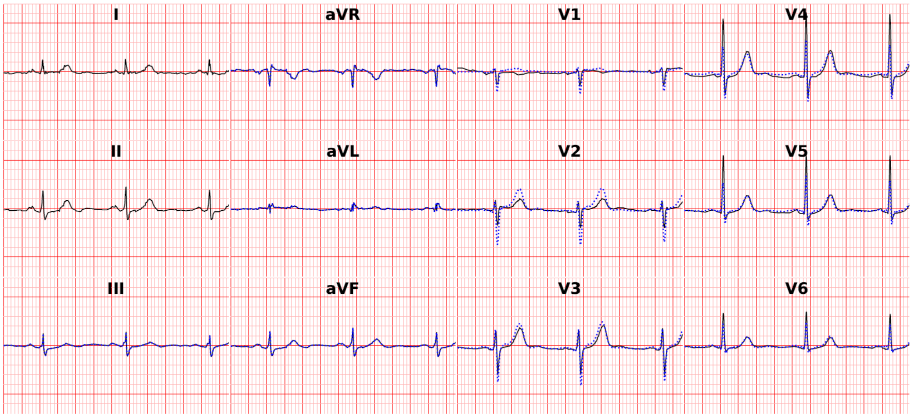

(b) 2-lead reconstruction

ECG plots depicting a real normal sample from the PTB-XL test set (black), alongside the reconstructed ECG (blue). The reconstructed ECG was generated using one lead (a) and two leads (b) with the generative adversarial network (GAN).

Supplementary Figure S3: Visualization of real and reconstructed electrocardiogram (ECG)

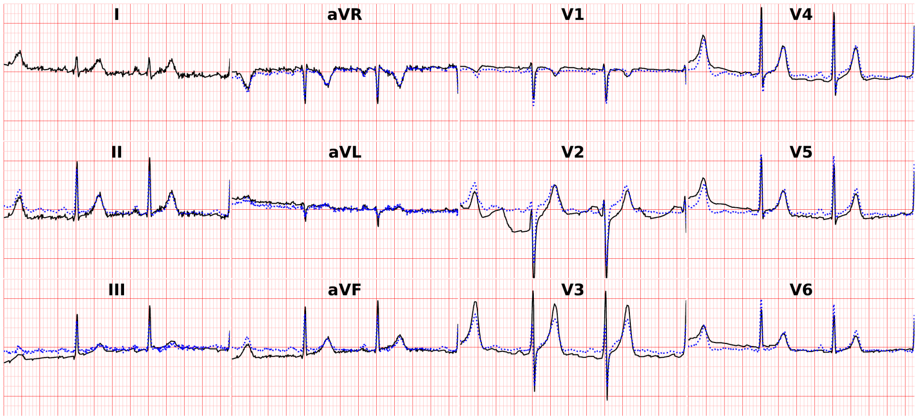

(a) 1-lead reconstruction

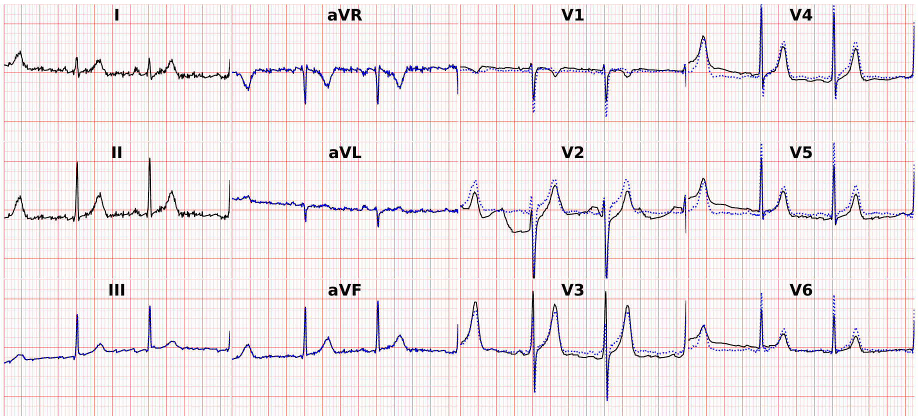

(b) 2-lead reconstruction

ECG plots depicting a real normal sample from the PTB-XL test set (black), alongside the reconstructed ECG (blue). The reconstructed ECG was generated using one lead (a) and two leads (b) with the generative adversarial network (GAN).

Supplementary Figure S4: Visualization of real and reconstructed electrocardiogram (ECG)

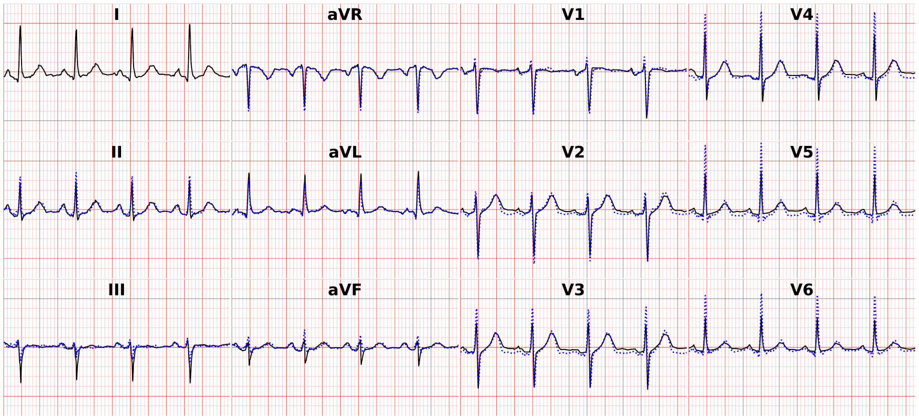

(a) 1-lead reconstruction

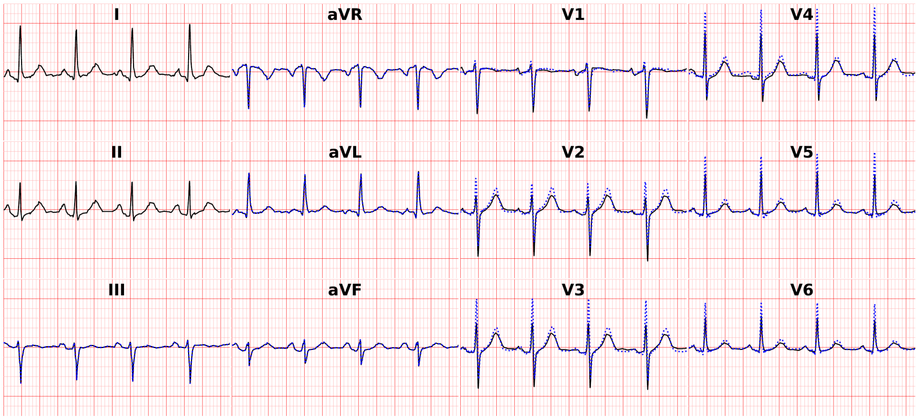

(b) 2-lead reconstruction

ECG plots depicting a real normal sample from the PTB-XL test set (black), alongside the reconstructed ECG (blue). The reconstructed ECG was generated using one lead (a) and two leads (b) with the generative adversarial network (GAN).

Supplementary Figure S5: Visualization of real and reconstructed electrocardiogram (ECG)

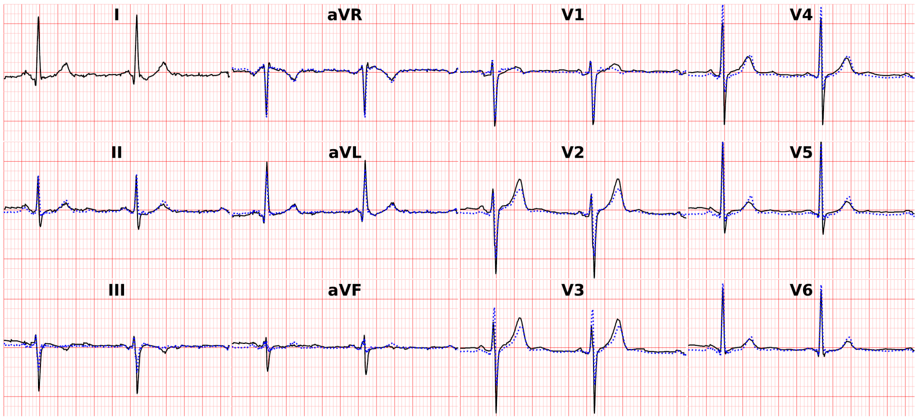

(a) 1-lead reconstruction

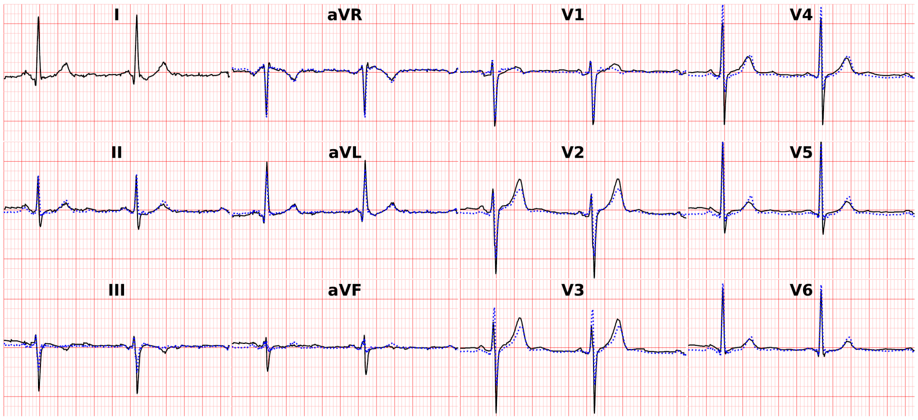

(b) 2-lead reconstruction

ECG plots depicting a real normal sample from the PTB-XL test set (black), alongside the reconstructed ECG (blue). The reconstructed ECG was generated using one lead (a) and two leads (b) with the generative adversarial network (GAN).

Supplementary Figure S6: Visualization of real and reconstructed electrocardiogram (ECG)

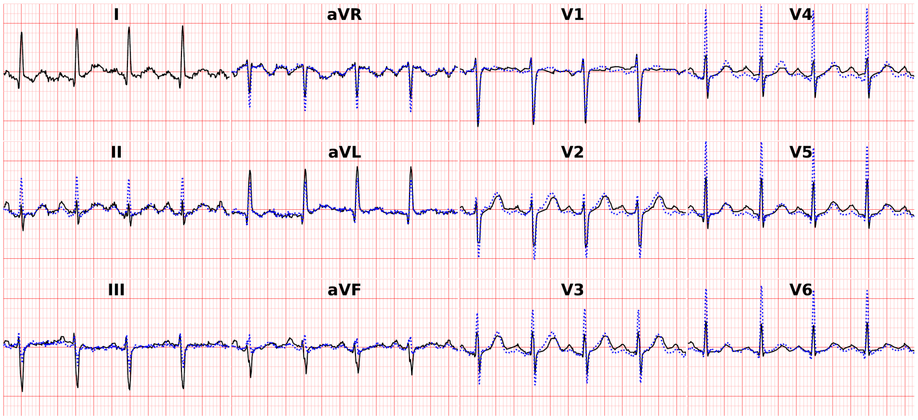

(a) 1-lead reconstruction

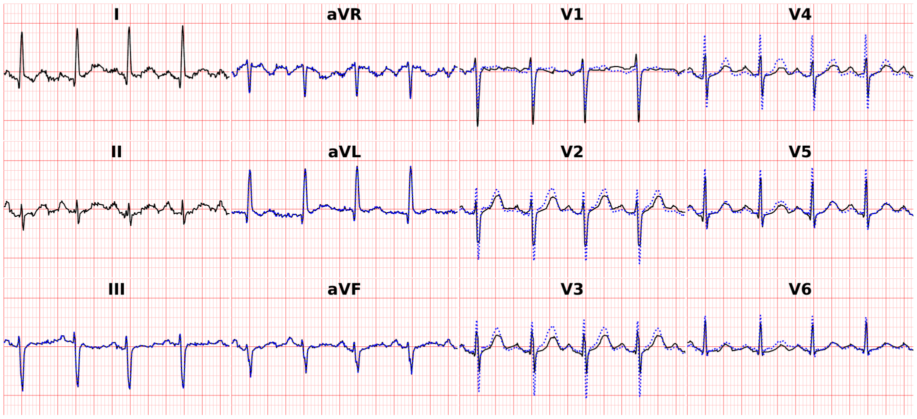

(b) 2-lead reconstruction

ECG plots depicting a real normal sample from the PTB-XL test set (black), alongside the reconstructed ECG (blue). The reconstructed ECG was generated using one lead (a) and two leads (b) with the generative adversarial network (GAN).
